# Perceived Body Image, Eating Behavior, and Sedentary Activities and Body Mass Index Categories in Kuwaiti Female Adolescents

**DOI:** 10.1155/2016/1092819

**Published:** 2016-11-30

**Authors:** Lemia H. Shaban, Joan A. Vaccaro, Shiryn D. Sukhram, Fatma G. Huffman

**Affiliations:** ^1^Department of Food Science and Nutrition, College of Life Sciences, Kuwait University, P.O. Box 5969, Safat, 13060 Kuwait City, Kuwait; ^2^Department of Dietetics and Nutrition, Robert Stempel College of Public Health and Social Work, Florida International University, Miami, FL, USA; ^3^Department of Biology, College of Staten Island, 2800 Victory Blvd, Office 6S-132, Staten Island, NY 10314, USA

## Abstract

*Background. *The State of Kuwait has a growing obesity epidemic in both genders and all age groups; however, obesity rates in the young seem to be rising.* Methods. *We conducted a cross-sectional survey in 169 Kuwaiti female adolescents attending both private and public schools spanning the six governorates in the State of Kuwait in order to explore female adolescents' self-image, body dissatisfaction, type of school (private versus public), TV viewing, and computer games and their relationship to body mass index.* Results.* Approximately half the students classified as obese perceived their body image to lie in the normal range. Females in the obese category were the most dissatisfied with their body image, followed by those in the overweight category. Eating behavior, level of physical activity, school type, television viewing, computer/video usage, and desired BMI were not significantly associated with level of obesity.* Conclusion. *This study was one of the few studies to assess adolescent females' body image dissatisfaction in relation to obesity in the State of Kuwait. The results suggest that including body image dissatisfaction awareness into obesity prevention programs would be of value.

## 1. Introduction

Obesity prevalence in Kuwait is primarily a result of the effects of modernization, affluence, increased food consumption, and sedentary lifestyles [1, 2]. Other factors that play a key role in escalating rates of obesity are sociocultural factors, biological factors, eating behaviors, hours of television viewing, and time spent on computers and video games [3–6]. Children living in the Middle East have one of the highest prevalences of overweight and obesity in the world, with Kuwait having the highest childhood overweight problem among the Gulf countries. Kuwait is also among the countries with the highest rate of obesity within the last 33 years (1980–2013), with the prevalence exceeding 50% of adult females [4, 7]. Several studies indicated that females present a greater overweight and obesity concern in Kuwait as opposed to males [7–10]. The higher prevalence of overweight and obesity in females may be attributed to genetic differences in fat storage; girls store more body fat than males in preparation for puberty and higher fat storage continues beyond puberty [11].

Westernization and nutrition transition have occurred at different rates in Arab countries [12, 13]. The movement toward Westernization has had a negative effect on health by promoting obesity [14] while thinness as a beauty standard is being promoted in the media [15]. Children are not resistant to media influences and girls, in particular, were observed to have body dissatisfaction as young as six years old [16–18]. Research suggests that girls' body image dissatisfaction, previously viewed as problems of adolescence, actually occurs long before puberty [19]. Although body dissatisfaction occurs in boys and girls who are overweight, it is more common in girls [20] and this can be seen later into adulthood where the prevalence of body image dissatisfaction is higher in women than in men in developing countries [21].

Perception of body image is an important factor in adolescents since overweight/obese adolescents are more likely to misperceive and underestimate their weight compared to those that are not overweight [22]. These perceptions may be partially attributed to role models: family members and also their immediate friends being overweight or obese. As a result, adolescents may have an inaccurate perception of what constitute appropriate weight status [22]. Underestimation of weight may serve as a protective factor for self-esteem; however, it can potentially lead to obesity-related chronic diseases [23]. Perception of body image is also crucial, because it is associated with a decreased quality of life in youth [24] and can directly predict a decrease in self-worth in some populations [25].

Type of school may also play a role in body image. Private as compared to public schools have been associated with a higher percent of child obesity among various African regions [26, 27]. A study done in the Seychelles indicated that the percent of overweight/obesity in children, aged 9–14, was significantly higher in private (29.0%) than in public (11.5%) schools [27]. Attending a private school may be associated with increased parental education such as in the Kingdom of Saudi Arabia [28] and Turkey [29]. The high prevalence of obesity in these nations has been attributed to greater purchasing power and leisure time which allows more time spent on sedentary recreational gadgets such as computers and computer games and prolonged hours of television viewing [26, 28].

The relationships among body image, diet, and sedentary behavior by school type have not been examined among Kuwaiti female school aged students aged 10–14; females were selected due to their higher rates of obesity and self-reported body dissatisfaction. Therefore, our objective was to investigate the interrelationships among obesity and obesity indicators: body image, sedentary activities, perceived physical activity, perceived diet, and school type in female adolescents.

## 2. Methods 

### 2.1. Study Design and Protocol

A total of 169 Kuwaiti female adolescents, aged 10–14, were selected from various schools in the State of Kuwait. Schools from the six governorates in Kuwait, with one private school and one public school per governorate, were included in the study sample in an effort to represent the diversity of the Kuwaiti student population. To ensure adequate power, sample size was based on a medium effect size for the chi-square test with one degree of freedom. A sample of 87 students was needed to achieve 80% power with an alpha of 0.05 (*N* = 151 for 6 degrees of freedom) [30].

### 2.2. Procedure

This study was approved by Kuwait University Institutional Review Board, the Ministry of Health, and the Ministry of Education. The Ministry of Health (Head of School Health Department) allocated two schools from each governorate, one public and the other private; they were selected for their representation of Kuwaiti students by the Ministry of Health. The Primary Investigator was then given the principals contact numbers in each school. The principals of the schools were contacted and a scheduled school visit was arranged. A nurse in each school was then assigned responsibility of allocating one classroom in each grade (grades 5–8) and distributing 20 questionnaires per grade. Classes were selected based on availability on the day that the consent forms were ready to be distributed to students; available classes were those that were not at the gym, science lab, computer lab, or taking an exam. We distributed a total of 80 questionnaires per school; more than triple the amount needed per school (*N* = 25) in an effort to compensate for any nonconsenting parents, no responses, or incomplete silhouette questionnaires. When class size was too small per grade, classes were merged. Available classes on the day of questionnaire distribution were given assent and consent forms to take home in order to allow students to participate in the study. Only those students who signed assent and whose parents signed informed consent were allowed to participate.

Upon consent, anthropometric measurements were obtained on predetermined days by a trained dietitian. Students were then asked to complete the silhouette questionnaire privately, without the presence of teachers or other students and with minimal assistance from the trained dietitians. Our initial goal of 300 participants was based on prior studies with approximately a sample size 300 [13, 31, 32] but this sample size was not reached due to the timeline of the study and participant response. The final sample size was 169 and data collection occurred from February to April 2015.

### 2.3. Measurements

Student's weight and height were measured with the subject standing erect without shoes, and, for weight, wearing light clothes using a portable Bearer GS 49_BMI clinical scale to the nearest 0.05 kg. All measurements were taken in triplicate to ensure accuracy. Body mass index (BMI) was calculated using the Centers for Disease Control and Prevention (CDC) zBMI calculator to compute BMI percentile [33]. Waist circumference to the nearest 0.1 cm was measured horizontally with a nonstretchable measuring tape placed midway between the 12th rib and iliac crest at minimal respiration to measure central obesity.

### 2.4. Silhouette Questionnaire

Our complete questionnaire was a modified form of the Barrett and Huffman questionnaire but was modified to suit the aims of the study [13]. The Stunkard figure rating scale [34] was used to assess body image perceptions, this scale consists of nine figures representing the most slender figure (rated as 1) to the heaviest figure (rated as 9), to assess body image perception. These scales were originally validated by Stunkard et al. in 1983 and have since shown good reliability in numerous populations including the Middle East and Kuwait [35–38]. The image was assigned to one of four categories using nine figure silhouettes, according to McElhone et al. [39]: pictures 1, 2, and 3 = underweight, pictures 4 and 5 = normal weight, pictures 6 and 7 = overweight, and pictures 8 and 9 = obesity.

The illustrations of female body shapes ranging from very thin to extremely obese were shown to the adolescents in school and they were asked to circle the female that looks most like them now (perceived), and the female that they wanted to look like (desired). They were also asked whether they considered their weight to be “Below Normal,” “Normal,” or “Above Normal.” The students were asked to select only one silhouette, which was coded from one (thinnest) to nine (heaviest). Additionally, questions related to physical activity and food habits such as “do you consider yourself a physically active person?”, “how many hours per week do you watch TV?” and “how do you rate your food habits?” were asked. Students' age, grade, name of school, date of birth, and school type (private or public) were also obtained during the questionnaire.

### 2.5. Major Study Variables

Demographics were obtained. Obesity was calculated as zBMI and measured by entering height, weight, gender, and age into the CDC's zBMI calculator to compute BMI percentile [33]. Three BMI categories were constructed based on the percentiles, where < 85th was considered underweight/normal; 85th–94th percentiles were classified as overweight; and ≥ 95th was classified as obese. Originally, we had categorized zBMI as four levels: underweight, normal weight, overweight, and obese and found *n* = 20 (11.9%) were underweight. We performed a cross-tabulation comparing these zBMI categories with body dissatisfaction level and found that 95% of the underweight category were satisfied with their current body image and only 5% were mildly dissatisfied. Due to the small number in these categories, we combined underweight with normal weight. Variables were tested for sufficient numbers in each category (>10%) and were collapsed when necessary. Body dissatisfaction was created by the difference between perceived BMI and desired BMI. Negative numbers were combined with zero for the lowest category of body dissatisfaction. A four-category variable allowed sufficient numbers in each cell. Eating behavior was determined by self-report using a single question, “Do you consider your diet to be healthy?” and responses were ordinal. The final variable was dichotomous (healthy/unhealthy) considering “most of the time/always” to be affirmative to healthy eating. Perceived physical activity was measured using a single question, “Do you consider yourself to be physically active?” and considered yes or no. Television viewing and computer/video time were asked as categorical variables in a range of hours per week. The final variables for television and computer/video were based on the 75th percentile and above as “high viewing” for television and “high usage” for computer/video. School type was converted to private or public based on the actual school attended.

### 2.6. Statistical Analysis

Relationships among obesity indicators were assessed using cross-tabulation and ordinal regression. General characteristics were run by zBMI categories and school type. Two ordinal regression models were run; one with category of zBMI percentiles and the other with category of body dissatisfaction. The independent factors included perceived eating behavior, perceived physical activity, television viewing, time on computer/video, and school type. Significance was considered at a *P* value of <.050. Statistics were performed using SPSS version 22.

## 3. Results

The general characteristics by BMI category are presented in Table 1. Two-thirds of students of normal BMI were satisfied with their body image, whereas only 30 and 12 percent were satisfied in the overweight and obese categories, respectively. Regardless of BMI, students wanted their BMI to look like silhouettes in the normal categories. Approximately half the students classified as obese perceived their body image to lie in the normal range, 23% perceived themselves overweight, and another 23% indicated that their body image was obese. Eighty percent of students with a normal BMI reported perceived engagement in physical activity, whereas a little more than half of those classified as overweight or obese considered themselves physically active. There were no significant differences across BMI categories for type of school, desired BMI, perceived eating behavior, television viewing, or computer/video usage.

Table 2 represents obesity indicators by type of school. Computer/video usage was higher at the 5–17.5 hours per week category for private school students as compared to public school students. However, this relationship was not linear and there were no significant differences for other usage times. Other obesity-related factors were not significantly different by school type.

Table 3 presents an ordinal regression model for level of body dissatisfaction with obesity factors. Considering the most dissatisfaction level, students in the lowest BMI percentile (<85th) were the least dissatisfied (ordered log odds) with their body image [*B* = −3.25 (−4.11, −2.38), *P* < .001] followed by those in the overweight category (85th–94th) [*B* = 1.59 (−2.48, −0.70), *P* < .001] as compared to students in the obese category (≥95th). Level of engagement in perceived physical activity, school type, television viewing, and computer usage were not significantly associated with body dissatisfaction. Perceived eating behavior was marginally significant: those reporting a fair to poor diet being more likely to have body dissatisfaction as compared to those reporting a good to excellent diet.

Ordinal regression for level of obesity with obesity factors is described by Table 4. The factors are compared to ascending level of obesity from zBMI scores in the normal range (<85th percentile) and the overweight range (85th–94th percentile) with obesity (≥95th percentile) as the reference. Students who perceived their BMI to be in the normal range and overweight range were less likely (ordered log odds) to be classified as obese [*B* = −2.57 (−3.85, −1.30), *P* < .001;* B* = −2.34 (−3.38, −1.30), *P* < .001], respectively. Perceived eating behavior, level of perceived physical activity, school type, television viewing, computer/video usage, and desired BMI were not significantly associated with level of obesity.

## 4. Discussion

Fifty percent of those classified as obese (32.2%) considered themselves to be of “normal” weight. This finding is in accordance with Maximova et al. [22] who reported of 24% overweight/obese children 1.6% perceived themselves overweight. Our finding may be due to family members and also their immediate friends being overweight or obese [22] or the effect of always being overweight which may alter children's perception of what is considered a normal BMI [40]. Kabir at al. [35] reported that underweight Kuwait University students were more dissatisfied with their body image than normal weight students. We did not have enough underweight participants to assess their body image. It should be noted that Kabir et al. [35] assessed young adults, whereas we studied adolecents; a shift in ideology may occur from adolescence to young adulthood and hence may explain our difference in findings.

There was a trend for private school adolescents to have a higher prevalence of overweight/obesity, although it was not significant (*P* = .302). A study in the Seychelles compared private and public schools, with the prevalence of overweight and obesity being significantly higher in private schools versus public schools (boys: 37% [31–44] versus 15% [14–16]; girls: 33% [26–41] versus 20% [19–22]) [27] and another study by Kyallo et al. [26] reported significantly higher overweight/obesity rates for private (29.0%) versus public (11.5%) school-age children aged 9–14 years in Kuwait. A plausible explanation as to why we did not find significance could be that Kuwaitis in both private and public schools have increased purchasing power regardless of school type. Private school parents may be more likely to work in the private sector where work hours are longer and more time-demanding than public sector jobs, which would allow greater time for students to remain sedentary at home and to snack freely without parent supervision.

Obesity was not associated with sedentary activities such as hours of television viewing and computer/video. Children who commonly view TV with commercials compared to those that watch TV without commercials had a higher BMI [41, 42]. Zimmerman and Bell [42] found that TV with commercials exposed children to advertising of food that is of poor nutritional quality, which in turn strongly influences children's food preferences and may subsequently alter their BMI. Concerning children's programs, Powell et al. [43] reported 97.3% of food and beverage products viewed in commercials by children 6–11 years of age did not meet the Interagency Working Group (IWG) for the recommended nutrients to limit (NTL) thus indicating uncontrolled exposure to the type of quality of commercials. In Kuwait, many families watch satellite TV where most of the children's channels have few commercials or are commercial-free; hence, these findings may explain why TV viewing was not significantly associated with obesity in our population.

A major strength of the study was that data were collected from all six governorates in the State of Kuwait in both private and public schools and it is the first study on school-age children addressing body image in relationship to their BMI. Limitations include omission of several confounders that could affect body image and obesity such as level of depression, type of television (with or without commercials), and level of perceived physical activity. Additionally, variables from questionnaires were by self-report, such as perceived diet and perceived physical activity, and may present bias. Moreover, nonparticipants may have different health beliefs as compared to those in the study. Finally, healthy eating was not objectively measured but was dependent upon how participants rated their food habits.

## 5. Conclusion

Strong public health initiatives are needed to address the growing obesity epidemic in Kuwait and our study highlighted the role and importance of self-perception. This study was one of the few to assess female adolescents' body image and image dissatisfaction with respect to obesity in the State of Kuwait. These findings highlight the importance of including education and counselling components concerning body image and image dissatisfaction in obesity programs for female adolescents.

## Figures and Tables

**Table 1:** General characteristics by BMI category.

Variable	<85th percentile Underweight/normal	85th–94th percentile Overweight	≥95th percentile Obese	*P* value
Body dissatisfaction (satisfied)	40 (67.8)^a^	8 (29.6)^b^	8 (11.9)^c^	<.001
Desired BMI (20.9)	27 (44.3)^a^	6 (22.2)^b^	34 (50.0)^a^	.052
Perceived BMI (23.1)	6 (10.2)^a^	6 (22.2)^a,b^	24 (34.8)^b^	<.001
Perceived engagement in physical activity (yes)	49 (80.3)^a^	15 (55.6)^b^	45 (64.3)^b^	.036
Perceived eating behavior (good-excellent)	30 (49.2)^a^	10 (37.0)^a^	35 (50.7)^a^	.511
Television viewing (3-4 hrs/wk)	20 (32.8)^a^	10 (37.0)^a^	16 (22.9)^a^	.208
Computer/video use (3–5 hrs/wk)	13 (21.3)^a^	6 (22.2)^a^	22 (31.4)^a^	.745
Type of school				
(public)	40 (65.6)^a^	18 (66.7)^a^	54 (77.1)^a^	.302
(Private)	21 (34.4)^a^	9 (33.3)^a^	16 (22.9)^a^	—

Data are *N *(%). Columns with the same letter have no significant differences.

**Table 2:** General characteristics by type of school (public versus private).

Variable	Public	Private	*P* value
Body dissatisfaction (satisfied)	39 (33.3)^^a^^	18 (39.1)^a^	.530
Desired BMI (20.9)	46 (38.7)^a^	25 (52.1)^a^	.279
Perceived BMI (23.1)	29 (24.4)^a^	9 (19.6)^a^	.439
Perceived engagement in physical activity (yes)	83 (68.6)^a^	34 (70.8)^a^	.776
Perceived eating behavior (good-excellent)	59 (49.2)^a^	25 (52.1)^a^	.864
Television viewing (3-4 hrs/wk)	35 (28.9)^a^	12 (25.0)^a^	.524
Computer/video use (5.5–17 hrs/wk)	22 (18.2)^a^	18 (37.5)^b^	.017

Data are *N* (%). Columns with the same letter have no significant differences.

**Table 3:** Ordinal regression model for body dissatisfaction.

Factors	Ordered log-odds: *B *(95% CI)	SE	*P* value
<85th % underweight/normal	−3.25 (−4.11, −2.38)	0.44	<.001
85th−94th overweight	−1.59 (−2.48, −0.70)	0.46	<.001
≥95th obese (reference)	0	—	—
Perceived fair to poor diet	0.65 (−.002, 1.30)	0.33	.051
Perceived good to excellent diet (reference)	0	—	—
Perceived engagement in physical activity (yes)	−0.02 (−0.73, 0.69)	0.36	.953
Perceived engagement in physical activity (no, reference)	0	—	—
Public school	0.46 (−0.27, 1.20)	0.38	.218
Private school (reference)	0	—	—
Hours/wk viewing television			
0–2.5	0.52 (−0.31, 1.74)	0.52	.170
3-4	0.55 (−0.44, 1.54)	0.41	.278
4.5–8.5	0.74 (−0.21, 1.69)	0.48	.128
>8.5 (reference)	0	—	—
Hours/wk using computer/video			
0–2.5	−0.04 (−1.04, 0.95)	0.51	.929
3–5	−0.39 (−1.35, 0.58)	0.49	.435
5.5–17.5	0.49 (−0.47, 1.46)	0.49	.316
>17.5 (reference)	0	—	—

Model fit: *χ* ^2^ = 79.4 (11), *P* < .001; Nagelkerke *R* ^2^ = 0.438.

**Table 4:** Ordinal regression for BMI percentile categories (underweight/normal, overweight, obese).

Factors	Ordered log odds: *B* (95% CI)	SE	*P* value
Perceived fair to poor diet	−0.45 (−1.14, 0.24)	0.35	.202
Perceived good to excellent diet (reference)	0	—	—
Perceived engagement in physical activity (yes)	−0.59 (−1.35, 0.17)	0.39	.129
Perceived engagement in physical activity (no, reference)	0	—	—
Public School	0.64 (−0.14, 1.41)	0.39	.107
Private school (reference)	0	—	—
Hours/wk viewing television			
0–2.5	−0.92 (−2.00, 0.15)	0.55	.093
3-4	−0.95 (−2.00, 0.09)	0.53	.072
4.5–8.5	−0.11 (−1.13, 0.91)	0.52	.833
>8.5 (reference)	0	—	—
Hours/wk using computer/video			
0–2.5	0.50 (−0.58, 1.58)	0.55	.367
3–5	0.75 (−.30, 1.80)	0.54	.161
5.5–17.5	0.16 (0.88, 1.19)	0.53	.764
>17.5 (reference)	0	—	—
Body Image:			
underweight/normal	−2.57 (−3.85, −1.30)	0.65	<.001
Overweight	−2.34 (−3.38, −1.30)	0.53	<.001
Obese	0	—	—
Desired BMI			
18.3	−0.51 (−2.01, 1.00)	0.77	.511
19.3	0.03 (−1.28, 1.35)	0.67	.961
20.9	0.24 (1.08, 1.56)	0.67	.720
23.1 (reference)	0	—	—

Model fit: *χ* ^2^ = 42.3 (14), *P* < .001; Nagelkerke *R* ^2^ = 0.274.
