# Corrigendum to “Perceived Body Image, Eating Behavior, and Sedentary Activities and Body Mass Index Categories in Kuwaiti Female Adolescents”

**DOI:** 10.1155/2017/3187290

**Published:** 2017-07-13

**Authors:** Lemia H. Shaban, Joan A. Vaccaro, Shiryn D. Sukhram, Fatma G. Huffman

**Affiliations:** ^1^Department of Food Science and Nutrition, College of Life Sciences, Kuwait University, P.O. Box 5969, Safat, 13060 Kuwait City, Kuwait; ^2^Department of Dietetics and Nutrition, Robert Stempel College of Public Health and Social Work, Florida International University, Miami, FL, USA; ^3^Department of Biology, College of Staten Island, 2800 Victory Blvd, Office 6S-132, Staten Island, NY 10314, USA

In the article titled “Perceived Body Image, Eating Behavior, and Sedentary Activities and Body Mass Index Categories in Kuwaiti Female Adolescents” [1], reference [13] was incorrectly cited instead of reference [23] in the sentence “our complete questionnaire was a modified form of the Barrett and Huffman questionnaire but was modified to suit the aims of the study [13]” in Section 2.4, “Silhouette Questionnaire.”
